# PKCepsilon and an increase in intracellular calcium concentration are necessary for PGF2alpha to inhibit LH-stimulated progesterone secretion in cultured bovine steroidogenic luteal cells

**DOI:** 10.1186/1477-7827-5-37

**Published:** 2007-08-30

**Authors:** Madhusudan P Goravanahally, Aritro Sen, Emmet K Inskeep, Jorge A Flores

**Affiliations:** 1 Department of Biology, West Virginia University, Morgantown, West Virginia, USA; 2 Department of Animal Sciences, Michigan State University, East Lansing, Michigan, USA; 3 Animal and Veterinary Sciences, West Virginia University, Morgantown, West Virginia, USA

## Abstract

The hypotheses that PKCepsilon is necessary for: 1) PGF2alpha to inhibit LH-stimulated progesterone (P4) secretion, and 2) for the expression of key prostaglandin synthesizing/metabolizing enzymes were tested in bovine luteal cells in which PKCepsilon expression had been ablated using a validated siRNA protocol. Steroidogenic cells from Day -6 bovine corpus luteum (CL) were isolated and transfected to reduce PKCepsilon expression after 48, 72 and 96 h. A third tested hypothesis was that an increase in intracellular calcium concentration ([Ca(2+)]i) is the cellular mechanism through which PGF2alpha inhibits luteal progesterone. The hypothesis was tested with two pharmacological agents. In the first test, the dose-dependent effects on raising the [Ca(2+)]i with the ionophore, A23187, on basal and LH-stimulated P4 secretion in cells collected from early (Day -4) and mid-cycle (Day -10) bovine CL was examined. In the second test, the ability of PGF2alpha to inhibit LH-stimulated P4 secretion in Day-10 luteal cells was examined under conditions in which an elevation in [Ca(2+)]i had been buffered by means of the intracellular calcium chelator, Bapta-AM.

PKCepsilon expression was reduced 65 and 75% by 72 and 96 h after transfection, respectively. In cells in which PKCepsilon expression was ablated by 75%, the inhibitory effect of PGF2alpha on LH-stimulated P4 secretion was only 29% lower than in the LH-stimulated group. In contrast, it was reduced by 75% in the group where PKCepsilon expression had not been reduced (P < 0.05). Real time PCR analysis indicated that there were no differences in the expression of cyclooxygenase-2 (COX-2), aldoketoreductase 1B5 (AKR1B5), prostaglandin E synthase (PGES), hydroxyprostaglandin-15 dehydrogenase (PGDH) and PGE2 -9-reductase as a function of PKCepsilon down-regulation. Finally, LH stimulated secretion of P4 at each luteal stage (Day -4 and -10), and PGF2alpha inhibited this only in Day -10 cells (P < 0.05). When A23187 was used at concentrations greater than 0.1 μmol, the induced elevation in [Ca(2+)]i inhibited the effect of LH on secretion of P4 in Day -4 and -10 cells (P < 0.05, Fig. 5). The inhibitory effect of PGF2alpha on LH-stimulated P4 in Day -10 cells was reduced if an increase in [Ca(2+)]i was prevented with Bapta-AM. These results support the hypothesis that differential expression of PKCepsilon and an elevation of [Ca(2+)]i are important for acquisition of luteolytic response to PGF2alpha.

## Background

The corpus luteum (CL) is a transient endocrine gland whose primary secretory product is progesterone (P4). The life span of the CL and consequently the amount of P4 it secretes is regulated according to reproductive physiological status. Substances reducing P4 secretion and shortening the luteal life span are said to be luteolytic [1,2].

In most species, including human beings, PGF_2_α is recognized as an important if not the main luteolytic factor [3-9]. During the ovarian cycle, the transition from early to mid-luteal phase is associated with changes in resistance/susceptibility to the luteolysin PGF_2_α; in cows, the CL is resistant to exogenous PGF_2_α prior to day 5 of the estrous cycle [10-17]. The cellular basis controlling luteal function during these physiological transitions, although studied intensely, is incompletely understood.

In steroidogenic cells of the ruminant CL, PGF_2_α activates its plasma membrane G-protein-coupled receptor, which in turn activates the membrane-bound phosphoinositide-specific phospholipase C (PLC), yielding inositol 1,4,5-trisphosphate (IP_3_) and diacylglycerol [18]. Indeed, in bovine luteal cells, PGF_2_α stimulated phosphatidylinositol 4,5-biphosphate hydrolysis and mobilized intracellular Ca^2+ ^[19]. Accordingly, calcium and protein kinase C (PKC) have been shown to be the intracellular mediators of PGF_2_β actions in luteal cells [20]. The regulatory effects of intracellular calcium concentration ([Ca^2+^]i) on progesterone might be biphasic as there is also evidence for a calcium requirement to support P4 synthesis by bovine luteal cells and LH, a luteotrophic hormone, increases IP_3_, and [Ca^2+^]i in bovine luteal cells and in porcine granulosa cells [21-23]. Therefore, there might exist thresholds of [Ca^2+^]i that support or inhibit P4 synthesis.

Choudhary et al, [17] tested the ability of increasing concentrations of PGF_2_α to increase the [Ca^2+^]i in large (LLC) and small (SLC) bovine luteal cells as function of development. Day-10 steroidogenic cells were more responsive to PGF_2_α than Day-4 cell. Response amplitudes and number of responding cells were significantly affected by agonist concentration, luteal development and cell type. Response amplitudes were greater in LLC than in SLC; responses of maximal amplitude were elicited with lower agonist concentrations from Day-10 than from Day -4 cells. Furthermore, on Day-10, as concentrations of PGF_2_α increased, larger percentages of SLC responded. Based on those results Choudhary et al proposed that the lower efficacy of PGF_2_α in the early CL was likely related to signal transduction differences associated with the PGF_2_α receptor at those two developmental stages [17].

The array of PKC isozymes expressed in whole bovine CL includes α, βI, βII, ε and μ[24-27]; and it has been demonstrated that the amount of PKCε expressed in the Day-10 CL is greater than in the Day-4 CL [26]. The latter observation led Sen et al, to propose that differential expression of PKCε as a function of development could play a role in the observed transitional resistance/susceptibility to PGF_2_α-induced luteal regression [26,27]. Sen et al, had further hypothesized that regulation of [Ca^2+^]i was a cellular mechanism through which PKCε could mediate actions of PGF_2_α on P4 secretion [27]. Additionally, there is evidence indicating that when bovine follicular theca cells are isolated and their luteinization is induced under in vitro tissue culture conditions, they express PKCδ [28]. As PKCδ has been reported to play an important role in other species such as in rabbits and rodents [29,30], this PKC isozyme might also be important for the physiology of the bovine ovary.

Endothelial cells of the bovine CL do not express PKCε, although they do express the other PKC isozymes described in the bovine CL [31]. Data obtained with Western blot and immunohistological assays indicated that steroidogenic cells are the main source of PKCε in the bovine CL [31]. Therefore, in experiment 1, in order to assess the potential physiological role of PKCε, we have used a siRNA strategy to down- regulate the expression of this PKC isozyme in luteal steroidogenic cells. In experiment 2, we used the PKCε down-regulated cells to test two hypotheses. Our first working hypothesis was that PKCε expression was necessary for PGF_2_α to inhibit LH-stimulated P_4 _secretion in vitro. The second working hypothesis was that PKCε was necessary for the expression of key genes of prostaglandin synthesis/metabolism that would favor PGF_2_α synthesis; whereas in PKCε down regulated cells, the expression of key genes of prostaglandin synthesis/metabolism would be such that synthesis of PGE2 would be favored. Finally, in experiment 3, we tested the hypothesis that [Ca^2+^]i is the cellular mechanism through which PGF_2_α inhibits luteal progesterone. We reasoned that if a pharmacological treatment is used to increase [Ca^2+^]i, this should inhibit luteal progesterone secretion with equally effectiveness, regardless of the developmental stage of the CL. Therefore, we used a pharmacological agent to increase [Ca^2+^]i and examine its effects on LH-induced P_4 _secretion in luteal cells collected from early (Day -4) and mid-cycle (Day -10) bovine CL. Furthermore, this hypothesis was also tested by using a pharmacological agent to buffer any increase in [Ca^2+^]i and examine, under conditions of low [Ca^2+^]i, the anti-steroidogenic effect of PGF_2_α on LH-induced P4 synthesis/secretion in cultures of luteal cells collected from mid-cycle (Day -10) CL.

## Methods

### Tissue collection

Non-lactating beef (experiments 1 and 2) or dairy (experiment 3) cows were observed visually for estrus twice daily at approximately 12-h intervals for a minimum of 30 min per observation. The day when standing estrus was observed was designated as Day 0 [32]. For experiments 1 and 2, the CL from four beef cows on Day-6 of the estrous cycle were collected in ice-cold saline and transported to the laboratory for luteal cell dispersion as described below. For experiment 3, 14 non-lactating dairy cows were synchronized with 25 mg PGF_2_α analog (Lutalyse^®^; Pfizer Animal Health., New York, NY) and ovaries on Day -4 (n = 4) or CL on Day -10 (n = 10) were collected surgically as described below and transported to the laboratory in ice-cold saline for dissociation and luteal cell enrichment as described below. The surgical procedure was performed via supravaginal incision under epidural anesthesia. For the epidural anesthesia, 6–9 ml 2% lidocaine were administered for cows weighing 450–700 kg (Butler Company, Columbus, OH). After surgery, penicillin (300,000 units) was administered intramuscularly to protect against post-surgical infection. The CL or ovary was collected into ice-cold phosphate buffered saline (PBS) at pH 7.4 and transported to the laboratory within 15 to 30 min after collection. The Animal Care and Use Committee of West Virginia University approved all procedures for these experiments (ACUC protocol # 06-0401).

### Luteal cell dispersion and purification

In the laboratory, the CL was dissected free of connective tissue, weighed, placed into cell dispersion medium (CDM, M-199 containing 0.1% BSA, 25 mM Hepes, 100 U/ml fungicide), and cut into small (about 1 mm^3^) fragments. The tissue fragments were processed for tissue dissociation as previously described [17]. Luteal endothelial cells were separated by a procedure previously described [17,33-35]. Briefly, magnetic tosylactivated beads (Dynal Biotech, Lake Success, NY) were used to separate endothelial cells and the non-adherent cells, steroidogenic-enriched luteal cells) were collected [33-35]. The cell population designated as steroidogenic cells represented a heterogeneous population of cells including fibroblasts, pericytes, lymphoid and possibly few endothelial cells not removed by the separation procedure. Cell viability and density were determined using Trypan Blue exclusion and a hemocytometer; luteal cell viability was usually greater than 96%.

#### Experiment 1

Validation of siRNA methodology for specifically down-regulating PKCε expression in enriched steroidogenic luteal cells.

Day-6 dissociated luteal steroidogenic cells were cultured overnight at a cell density of 1 × 10^6 ^cells/well in 35 mm 24 – well culture dishes (Corning Inc, Corning NY) containing 1 ml Medium 199 supplemented with 5% fetal calf serum (FCS, GIBCO) at 37°C (95% air, 5% CO^2^). The next day cells were transiently transfected with PKCε-specific siRNA kit (Upstate Cell Signaling solutions, Lake Placid NY) using lipofectin 2000 kit (Invitrogen Life Technologies) following the procedure recommended by the manufacturer. After transfection for 4 hr, the cultures were provided with M199 supplemented with 10% FCS, and incubated for a total of 48, 72 or 96 hours. After each of these time points, the cells were collected by adding 2 ml M199 containing 0.25% trypsin (GIBCO) to cover the monolayer and leaving the culture dish for about 1 min at room temperature. The cells were aspirated and washed one time with M199 containing 5%FCS and once with M199 without FCS. Cells collected from duplicate wells were pooled and the efficiency of transfection at 48, 72 and 96 h was analyzed by RT-PCR and Western blot analysis. Control groups included cells cultured in presence of M199 alone, M199 and transfecting reagent, and cells treated with non-specific siRNA duplex (non-specific siRNA).

#### Experiment 2

Effects of down-regulating PKCε expression by the siRNA protocol on: A) the ability of PGF_2_α to inhibit the LH-stimulated P4 accumulation, and B) on the expression of key genes involved in prostaglandin synthesis and metabolism.

*Hypothesis 1*: PKCε is necessary for PGF_2_α to be able to inhibit P_4 _secretion. To examine the ability of PGF_2_α (Cayman Chemical, Ann Arbor, MI) to inhibit LH-induced progesterone accumulation, the siRNA transfected and control cells (not treated with PKCε siRNA) were treated, after 96 h, with100 ng/ml of LH, 1000 ng/ml of PGF_2_α, or a combination of LH and PGF_2_α for 4 hrs. After this time, the cell free medium was collected from each treatment and frozen until determination of P4 by radioimmunoassay (RIA). The RIA used for measurements of P4 in the culture media has been described previously [36]. The standard curve for this RIA ranged from 10 pg/ml to 800 pg/ml, and the intra- and interassay coefficients of variation were 9.2% and 12.8%, respectively.

*Hypothesis 2*: PKCε is necessary for the expression of key prostaglandin biosynthetic/metabolizing enzymes. For the real time quantitative determination of gene expression of key prostaglandin biosynthetic/metabolizing enzymes in PKCε down-regulated and control (not down-regulated) cells, RNA samples were obtained from the cells collected in the experiment described under Hypothesis 1. The genes examined were: aldoketoredutase 1B5 (AKR1B5), prostaglandin-15 dehydrogenase (PGDH), prostaglandin E synthase (PGES), prostaglandin E-2-9-redutase, and cyclooxygenase-2 (COX-2). Enriched steroidogenic cells were treated with the PKCε siRNA protocol and after 96 h of culture the cells were treated with LH (100 ng/ml), PGF_2_α (1000 ng/ml), or a combination of LH and PGF_2_α for 4 h. The cells were collected by a brief trypsin treatment and total RNA was isolated with Trizol reagent according to the manufacturer's instructions (GIBCO). Total RNA was quantified spectroscopically at 260 nm and integrity of the RNA was determined by 2% agarose gel electrophoresis. Specific primers were designed by using primer 3 software. The primer sequences and their accession numbers are shown in table 1. The single-step RT-PCR was carried out and cDNA product for each gene was column purified. Ten-fold serial dilutions of cDNA for each of the genes were used as templates to generate standard curves. Total RNA samples were reverse transcribed and used as templates in an iQ5 cycler (Bio-Rad Laboratories, Hercules, CA). The 25 μl reaction mixture contained 12.5 μl SYBER green mix (Bio-Rad Laboratories), 2 μl cDNA sample, 2.5 μl each sense and antisense primers (0.5 μmol) and 5.5 μl of RNAse -free H_2_O. The standard curves of threshold cycle (ct value) versus log starting quantity for the genes of interest were obtained. The conditions used were as follows: inactivation of RT enzyme, 95°C/3 min; denaturation, 95°C/30 sec; annealing, 55°C/30 sec; and extension, 72°C/1 min with fluorescence acquisition. The melt-curves were generated from 55°C to 95°C with 0.5°C increments in temperature. The melt-curves were observed for presence of single amplification product. The slope and intercept values obtained from the standard curve were used to determine the starting quantity for each gene using linear regression equation and gene expression for the desired gene was normalized using β-actin as the reference gene.

**Table 1:** Primer sequence, accession number, product size and annealing temperature of investigated genes

Gene	Primers	Acc#	Size	Annealing
β-Actin	F5'GACATCCGCAAGGACCTCTA3' R5' ACGGAGTACTTGCGCTCAG3'	BC102948	100	*
PGDH	F5'GGAAAGCTGGACATCTTGGT3' R5'GCAAATTGCGTTCAGTCTCA3'	BC102458	150	60°C
PGES-1	F 5'GAACGACCCAGATGTGGAA3' R5'ATACGGCCCAGGAAGAAGAC3'	NM_174443	153	59°C
AKR1B5	F 5' GACCTTGGGTACCGTCACAT3' R5'TCTTTCTCACTGGGAATCACG3'	S54973	150	59°C
PGE2 9- Reductase	F 5'AAGAAATGCAGCCGTGAACT3' R 5' GCTCCTTCTTCTGGGCTTTT 3'	BC102943	155	59°C
COX-2	F5'CATGATGTTCTTTGTTGGCATT3' R 5' GCGAATTCCAACTTTCCATC3'	AF031698	154	60°C

• Actin was amplified under the same conditions as gene of interest

#### Experiment 3

The working hypothesis was that a rise in [Ca^2+^]i is the cellular mechanism through which PGF_2_α inhibits luteal P4.

*Effect of a pharmacological increase in [Ca^2+^] i on the LH-stimulated P4 synthesis/secretion in Day-4 and -10 luteal steroidogenic cells*. We predicted that if [Ca^2+^]i is increased by a pharmacological treatment, this increase in [Ca^2+^]i should be equally effective in reducing the LH-stimulated P4 secretion regardless of the developmental stage of the CL. The enriched steroidogenic cells (1 × 10^5 ^cells/well) isolated from Day-10 and Day-4 CL of PGF_2_α-synchronized non-lactating dairy cows were cultured overnight in 15 mm 24 -well culture plates in medium M199 supplemented with 0.1% BSA and 0.5% FCS. The next morning, the cells were treated in duplicate wells for 24 hr with M199 (control), LH (100 ng/ml), PGF_2_α, (1.0 μg/ml), and a combination of LH and PGF_2_α. The ability of increasing concentrations of the calcium ionophore, A23187 (0.1, 1, 10, or 100 μmol, (Invitrogen Detection Technologies), to inhibit basal and LH -stimulated P4 synthesis/secretion was tested in duplicate wells. The medium for the control group contained 0.1% dimethylsufoxide (DMSO, Pierce Rockport, IL), the solvent used for PGF_2_α and A23187. The cell-free media were collected and frozen until later measurements of P4 by RIA. The concentrations of A23187 used were based on single-cell studies, in which a concentration of 1 μmol A23187 was usually effective in increasing [Ca^2+^]i to values comparable to those seen when cells were stimulated with PGF_2_α at a concentration of 1000 ng/ml. The concentration range used of the Ca^2+ ^ionophore should assure a very good probability of eliciting a wide range in increases in [Ca^2+^]i that would allow testing its effect on the LH-stimulated P4 synthesis/secretion in Day-4 and -10 steroidogenic cells.

*If the PGF*_2_α *-stimulated increase in [Ca*^2+^*]i is prevented, PGF*_2_α *T will not be able to inhibit P4 synthesis/secretion*. This experiment examined the ability of PGF_2_α to inhibit LH-stimulated P4 secretion in Day-10 luteal cells under conditions in which elevations in [Ca^2+^]i were buffered. This was accomplished by testing the effect of 1,2-bis(2-aminophenoxy)ethane-N, N, N', N'-teyracetic acid tetrakis acetomethyl ester, Bapta-AM (Invitrogen Detection Technologies, Carlsbad, CA), an effective pharmacological agent known to buffer changes in [Ca^2+^]i [37,38]. The concentration range chosen, 0.1 to 1000 μmol, was based on preliminary single-cell studies indicating that at the concentration of 10 μmol, Bapta-AM effectively prevented the typical increase in [Ca^2+^]i induced by PGF_2 _in luteal steroidogenic cells. The enriched Day-10 steroidogenic cells (1 × 10^5 ^cells/well) isolated as described above were cultured overnight in 15 mm 24 -well culture plates in medium M199 supplemented with 0.1% BSA and 0.5% FCS. The next morning, the cells were treated in duplicate wells for 24 hr with M199 (control), LH (100 ng/ml), PGF_2_α, (1.0 μg/ml), and a combination of LH and PGF_2_α with increasing concentration of Bapta-AM (0.1, 1, 10, 100 or 1000 μmol). The effect of each treatment on basal and LH -stimulated P4 synthesis/secretion was tested in duplicate wells. The medium for the control group contained 0.1% dimethylsufoxide (DMSO, Pierce Rockport, IL), the solvent used for PGF_2_α and Bapta. The cell-free media were collected and frozen until later measurements of P4 by RIA.

### Semi-quantitative RT-PCR

The time-course effectiveness of the siRNA treatment in down-regulating PKCε mRNA expression was determined by a semi-quantitative RT-PCR procedure (RT-PCR, Qiagen, Valencia, CA) previously validated and described [24]. In this RT-PCR assay, PKCε expression was normalized to the expression of GAPDH as the reference gene. The sequence of the PKCε and GAPDH primers were those previously published: [17], sense 5'-AGCTTGAAGCCCACAGCCTG-3'; antisense 5'-CTTGTGGCCGTTGACCTGATG-3'; and (34), sense 5'- TGTTCCAGTATGATTCCACCC-3'; antisense 5'- TGTTCCAGTATGATTCCACCC-3' respectively. The specificity for these primer sets have been documented (17, 24), and confirmed here by using the nucleotide database of National Center for Biotechnology Information [39] with BLAST software. The RT-PCR assay conditions were as follows: 50°C for 30min for reverse transcription reaction, 95°C for 15min for inactivation of RT enzyme, and then for PCR cycles consisted of 95°C for 50seconds for denaturing, 58°C for 30seconds for annealing, 72°C for 1min for extension and a final extension of 5min at 72°C. The RT-PCR products were electrophoresed on 2% agarose gel stained with ethidium bromide and viewed using the Fluro-S MultiImager (Bio-Rad Laboratories). Data were collected using densitometric analysis of Quantity One quantification software package (Version 4, Bio-Rad Laboratories). The intensity of the signal corresponding to PKC isozyme was standardized by the corresponding intensity of GAPDH control in that sample.

#### Semi-quantitative Western blots

Proteins were isolated from cells of siRNA treated and control groups using previously described methodology [24]. Details for the semi-quantitative Western blot protocol used here have been described elsewhere [26]. Briefly, protein samples (10 μg/lane) were resolved on an 8% polyacrylamide gel. The resolved proteins were transferred to polyvinylidene fluoride membrane (Biotechnology Systems, Boston, MA). The membranes were treated for immunodetection of the proteins of interest. The following primary antibodies were used: a mouse anti-actin monoclonal antibody ([used at a dilution of 1:3000 (v/v] Chemichon International, Inc., Temecula, CA); PKC isozyme specific (α, βI, βII, ε,) polyclonal antibodies and their antigenic peptides ([antibodies used at dilution 1:1000] Gibco, Grand Island, NY). The following horseradish peroxidase-conjugated secondary antibodies were used here: anti-rabbit (1:5000, v/v; Amersham Pharmacia Biotech, and anti-mouse (1:30,000 v/v; GIBCO). Densitometry of the bands of interest were performed using Quantity One quantitation software. The intensity of the signal corresponding to the protein of interest was standardized by the corresponding intensity of the actin control in that sample. This normalization of data allows an estimate, in a semi quantitative manner, the amount of protein in the samples of interest, as described earlier [26].

### Statistics

The statistical software program from Statistical Analysis System, JMP 3.0 was used for data analyses [40]. Data were expressed as means ± SEM for all the experiments. One-way ANOVA was used to determine effects of different treatments. Tukey – Kramer HSD was used to compare the different treatments subgroups. A value of P < 0.05 was considered statistically significant.

## Results

### Experiment 1

Culturing steroidogenic cells collected from the Day -6 CL spontaneously induced the expression of PKCε (data not shown). Expression of PKCε was induced gradually by the tissue culture conditions, and as Day-6 luteal cells were cultured up to 6 days, PKCε expression had been spontaneously increased to values comparable to those seen in Day-10 CL (data not shown).

**Figure 1:**
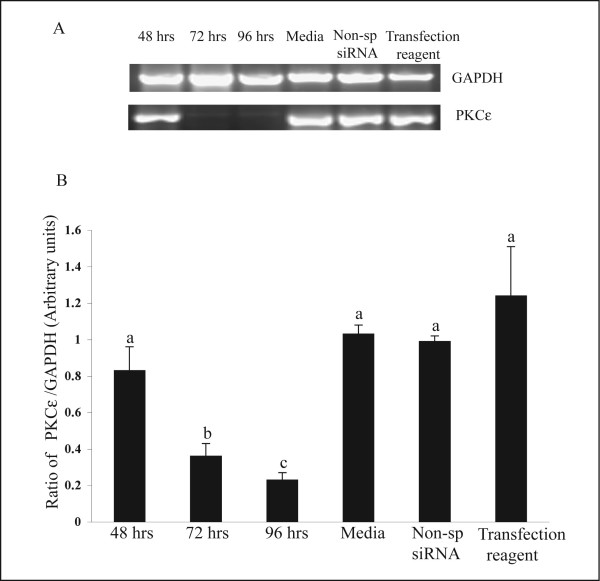
Time-course reduction in PKCε mRNA expression after transfection of luteal steroidogenic cells with PKCε specific siRNA. (**A**) Representative RT-PCR products obtained from total RNA using the PKCε and GAPDH primers. The amount of total RNA was adjusted to 200 ng per reaction and 40 cycles were used for PKCε ; while 28 cycles were used for GAPDH. The size of the amplified products for the GAPDH and PKCε were 900 and 500 bp, respectively. PKCε and GAPDH mRNA expression after 48, 72, and 96 h of transfection with PKCε specific siRNA are shown. Lanes labeled Media, non-specific (Non-sp) siRNA, and Transfection reagent represent respective treatments without PKCε specific siRNA treatment. GAPDH was used as the control gene to normalize the PKCε mRNA expression. (**B**) Quantitative analysis of the RT-PCR products obtained in four (n = 4) replicates similar to those shown in panel A. Data are the mean mean ± SEM of the densitometry measurements for PKCε relative to GAPDH mRNA. Statistical comparisons were made between different treatments. Different letters above each SEM represent different values (P < 0.05).

Fig. 1A shows a typical result of the time-course siRNA experiments performed. The summarized data shown in Fig. 1B indicate that there was a significant (P < 0.05) decrease in the amounts of mRNA encoding PKCε after 72 h of transfection (0.36 ± 0.07) compared to the media- treated control group (1.03 ± 0.05). Panels A and B in Fig. 1 show that this approach reduced PKCε expression 65 and 75% (0.23 ± 0.04) by 72 and 96 hrs of treatment respectively. This reduction was specific because no similar changes were observed in treatments receiving only experimental media (Media), receiving non-specific siRNA duplexes (Non-Sp siRNA), or receiving only transfection reagents (Transfection reagent, Fig. 1B).

**Figure 2:**
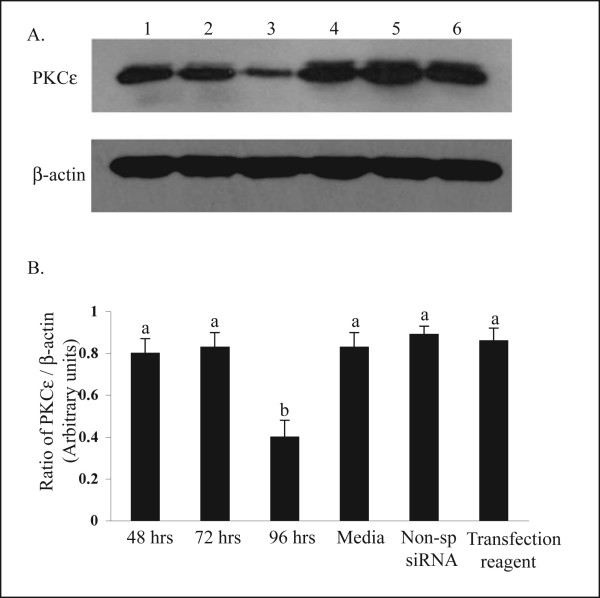
Reduction in PKCε protein. (**A**) Representative Western blot showing the amount of PKCε and actin expressed in protein samples prepared from luteal steroidogenic cells after 48, 72, and 96 h of transfection with PKCε specific siRNA (lanes 1–3). Lanes labeled 4 – 6, contained protein samples from indicated control treatments (media, Non-sp siRNA, and transfection reagent, respectively). (**B**) Semi-quantitative analysis of the densitometry derived from four experiments similar to the one shown in panel A. the y-axis shows the ratio of the optical density ratio of PKCε to that of its corresponding β-actin. The data are shown as mean ± SEM, and comparisons were made between different treatments. Values with different letters denote differences by one-way ANOVA followed by Tukey-Kramer honestly significant difference (P < 0.05).

**Figure 3:**
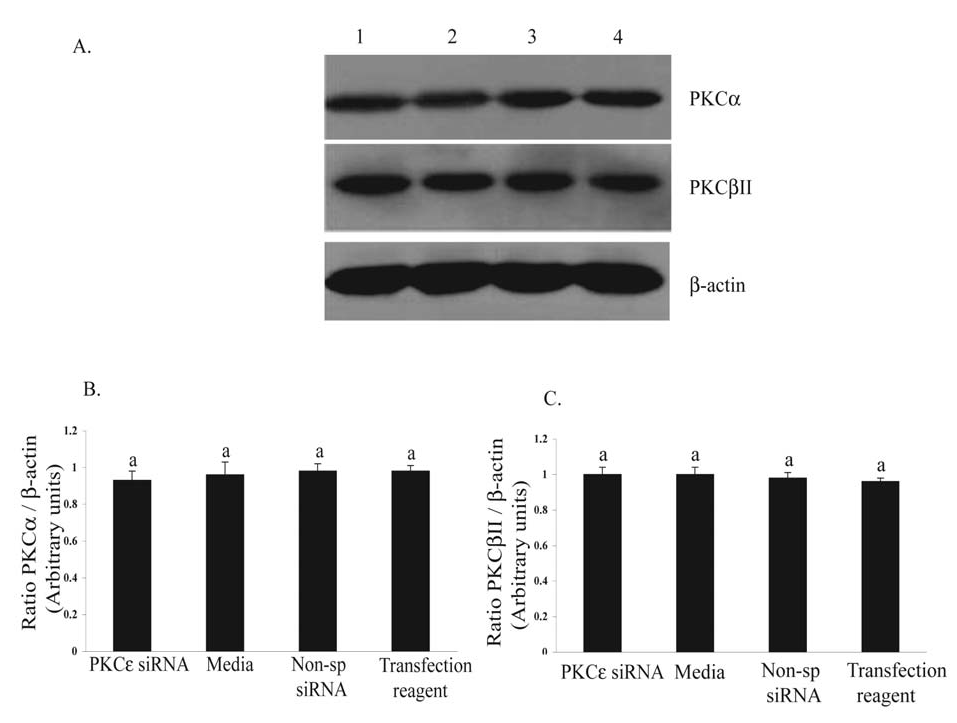
PKCα and PKCβII protein after 96 h transfection of luteal steroidogenic cells with PKCε specific siRNA. (**A**) Representative Western blot showing the amount of PKCα, PKCβII KCε and actin detected in protein samples prepared from luteal steroidogenic cells after 96 h of transfection with PKCε specific siRNA (lane 1). Lanes labeled 2 – 4, contained protein samples from indicated control treatments (media, Non-sp siRNA, and transfection reagent, respectively. **B and C**) Semi-quantitative analysis of the densitometry derived from four experiments similar to the one shown in panel A for PKCα (**B**) and PKCβII (**C**). The y-axis shows the ratio of the optical density ratio of PKC isozyme to that of its corresponding β-actin. The data are shown as mean ± SEM, and comparisons were made between different treatments by one-way ANOVA followed by Tukey-Kramer honestly significant difference.

The effectiveness of the siRNA transfection in reducing protein corresponding to PKCε can be seen in the semi-quantitative western blotting (Fig 2A). A visual reduction in protein was detected 72 h after transfection (Fig. 2A). However, the semi-quantitative analysis of the data indicated that a significant reduction (P < 0.05) in the amount of PKCε protein had not occurred until 96 h after transfection. At this time, there was a 50% reduction in the siRNA -treated group (0.39 ± 0.02) compared to control group (0.82 ± 0.07, Media, Fig. 2B). Figure 3 demonstrates the specificity of the siRNA transfection in down-regulating the PKCε isozyme. This Western blot was carried out for other PKC isozymes, PKCα and PKCβ II, and there was no reduction in the amounts of these isozyme proteins even at 96 h after transfection with PKCε-specific siRNA; a time by which there was significant reduction in PKCε (Fig. 2B).

### Experiment 2

**Figure 4:**
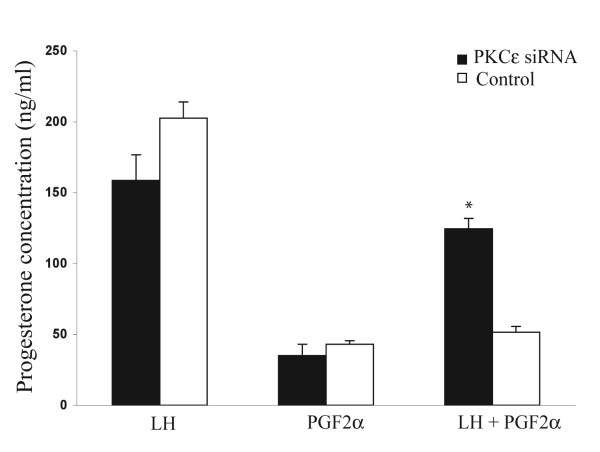
Effects of PKCε down-regulation on the ability of PGF_2_α to inhibit the LH-stimulated progesterone synthesis/secretion in cultures of steroidogenic luteal cells transfected for 96 h with PKCε specific siRNA (filled bars) or with transfection regents (control, open bars). Progesterone accumulation was determined in culture media after 4 h of incubation in the following treatments: LH (100 ng/ml), PGF_2_α (1 μg/ml) and a combination of PGF_2_α and LH. Data are presented as mean ± SEM of four individual replicates (n = 4 cows). For each treatment group, statistical comparisons were made between PKCε down-regulated (PKCε siRNA) and control (not PKCε down-regulated); different letters above each SEM denote different values, P < 0.05.

*Hypothesis 1: Effect of PKCε down-regulation on the ability of PGF*_2_α *to decrease the LH-induced P4 accumulation*. Enriched steroidogenic cells (n = 4) transfected with PKCε siRNA were cultured for 96 h and treated with LH, PGF_2_α, and combination of LH and PGF_2_α for 4 h. The control group included cells treated with the hormones described above, but expressing normal amount of PKCε. PKCε down-regulation did not induce a decrease in the amount of P4 accumulation in the LH-stimulated cells (158.4 ± 18.1) compared to the control (202.4 ± 11.4). As in previous experiments, the accumulation of P_4 _was significantly reduced (P < 0.05) by PGF_2_α (42.9 ± 2.6) compared to LH -treated control group (202.4 ± 11.4). There was no difference in the amounts of accumulated P4 between PGF_2_α-treated PKCε down-regulated cells (34.9 ± 8.1) and control group (42.9 ± 2.6). However, the ability of PGF_2_α to decrease LH-stimulated P4 accumulation was significantly (P < 0.05) inhibited in the PKC ε down-regulated group, 124.4 ± 7.4 compared to control, 51.4 ± 4.1 (Fig. 4).

**Figure 5:**
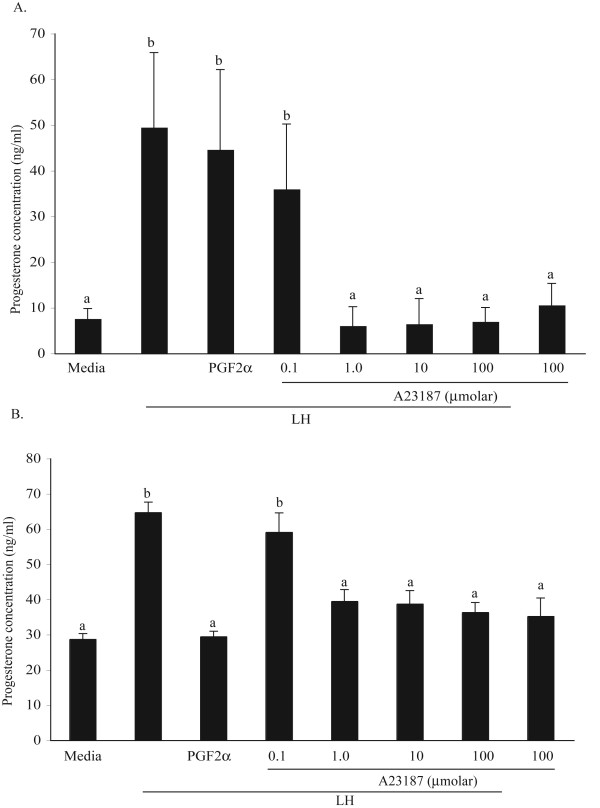
Effect of the Ca^2+ ^ionophore, A23187, on basal and LH-stimulated progesterone synthesis/secretion (ng/ml) in cultured steroidogenic cells collected from Day 4 (panel A) and Day 10 (panel B) bovine CL. Progesterone accumulated in culture media was determined after 4 h of incubation in the following treatments: media alone (Media), LH (100 ng/ml), LH and PGF_2_α (1000 ng/ml), or LH and A23187 (0.1, 1, 10, and 100 μmol). As explained in Materials and Methods, these treatments also contained 0.1% of the solvent used for PGF_2_α and A23187, DMSO. Data are presented as the mean ± SEM of four Day 4 and 10 Day 10 individual replicates (n = 4 and 10 cows respectively). Statistical comparisons were made across treatments, and means with different letters, differ within each panel (P < 0.05).

*Hypothesis 2: Gene expression of key prostaglandin biosynthetic/metabolizing enzymesin PKCε down-regulated cells*. Real-time PCR analysis of total RNA for mRNA encoding Cox-2, AKR1B5, PGES, PGDH and PGE (2) -9-ketoreductase indicated that there were no significant differences in the expression of any of these genes as a functions of PKCε down-regulation, LH or PGF_2_α treatment (data not shown).

### Experiment 3

*A rise in [Ca*^2+^*]i is the cellular mechanism through which PGF*_2_α *inhibits luteal P4*.

*Effect of a pharmacological increase in [Ca^2+^]i on the LH-stimulated P4 synthesis/secretion in Day-4 and -10 luteal steroidogenic cells*. As reported in previous studies [17], basal P4 accumulation in cells collected form Day -4 CL was significantly lower than in those collected from Day -10 (7.6 ± 2.2 and 29.2 ± 1.8 respectively, Fig. 5A and 5B). LH significantly increased (P < 0.05) the luteal progesterone accumulation in both Day- 4 (49.5 ± 16.3) and -10 cells (65.7 ± 3.7). This effect of LH was not inhibited by PGF_2_α in Day- 4 cells (44.6 ± 17.5), whereas it was significantly inhibited in Day-10 cells (31 ± 1.9, Fig. 5A and 5B). When used at 0.1 μmol, A23187 did not reduce LH-stimulated P4 accumulation in Day -4 or -10 cells; but at higher concentration (1.0 – 100 μmol), it negated the stimulatory effect of LH on P4 (P < 0.05, Fig. 5A – B). Basal P4 accumulation in Day -4 and -10 cells was not affected by any concentrations of A23187 tested (Fig. 5A and 5B, only 100 μmol A23187 shown).

**Figure 6:**
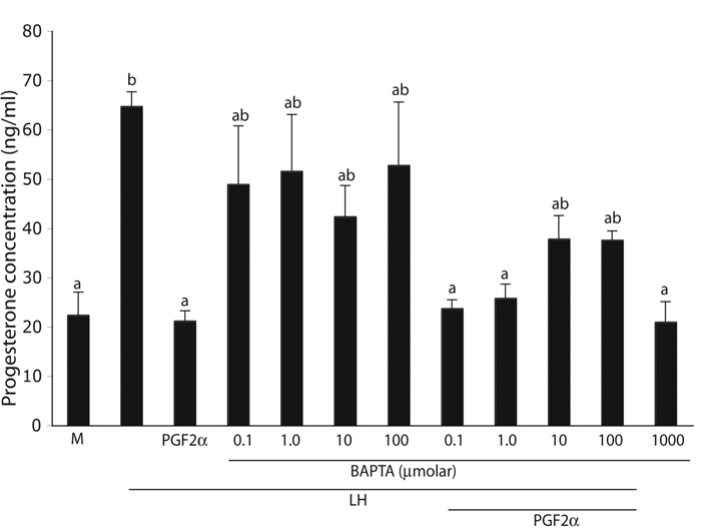
Effect of the cell-permeable calcium chelator, Bapta-AM, on basal and LH-stimulated progesterone synthesis/secretion (ng/ml) in cultured steroidogenic cells collected Day 10 bovine CL. Progesterone accumulated in culture media was determined after 4 h of incubation in the following treatments: media alone (Media), LH (100 ng/ml), LH and PGF_2_α (1000 ng/ml), or LH and Bapta-AM (0.1, 1, 10, and 100 μmol). As explained in Materials and Methods, these treatments also contained 0.1% of the solvent used for PGF_2_α and Bapta-AM, DMSO. Data are presented as the mean ± SEM of four Day 10 individual replicates (n = 4 CL obtained from 4 cows). Statistical comparisons were made across treatments, and means with different letters denote different values, P < 0.05.

*If the PGF*_2_α *-stimulated increase in [Ca*^2+^*]i is prevented, PGF*_2_α *T will not be able to inhibit P4 secretion*. LH significantly increased (P < 0.05) the luteal progesterone accumulation in Day- 10 cells (64.6 ± 3, Fig. 6). This effect of LH was completely inhibited by PGF_2_α (21.1 ± 2.1, Fig. 6). Importantly, basal P4 accumulation (Fig. 6) was not affected by the Bapta-AM treatment, not even the highest concentration used (20.9 ± 4.1). When Bapta-AM was used at 0.1, 1, 10 and 100 μmol in combination with LH, the values on P4 accumulation became intermediate between those observe for basal and LH alone (Fig. 6); and the stimulatory effect of LH was completely eliminated by 1000 μmol Bapta (data not shown). Consequently, the effect of Bapta-AM on the anti-steroidogenic action of PGF_2_α could only be tested up to 100 μmol. The inhibitory effect of PGF_2_α on LH-stimulated luteal P4 accumulation was not affected by Bapta when used at concentrations not exceeding 1 μmol, as the values for P4 accumulation clearly were not different from those observed for basal values (P < 0.05, Fig. 6). However, at 10 and 100 μmol, Bapta-AM effectively reduced the ability of PGF_2_α to inhibit the stimulatory effect of LH on P4 accumulation (Fig. 6).

## Discussion

The roles of specific PKC isozymes in luteal physiology have received little attention to date. As discussed below, these studies were designed to test the effects of ablating PKCε expression in order to examine its hypothesized function. Previous studies had indicated that a potential function for PKCε might be to regulate quantitatively the intracellular calcium signal initiated by PGF_2_α on one of its luteal targets, the steroidogenic cells. The present studies validate the effective and specific down-regulation of PKCε by siRNA technology and provide strong evidence about the function of this PKC isozyme in luteal physiology. The data support the overall hypothesis that down-regulating expression of PKCε reduces the effectiveness of PGF_2_α in reducing progesterone synthesis/secretion. This observation extends the report that when PKCε was inhibited with PKCε-specific inhibitors, the PGF_2_α – induced rise in [Ca^2+^]i was decreased in LLC and SLC and that this in turn had consequences (at least in part) in the ability of PGF_2_α to inhibit LH-stimulated P4 secretion at this developmental stage [27]. As previously reported [17], LH induced an increase in the amount of P4 secretion. Interestingly, in the group where PKCε expression was down -regulated, the inhibitory effect of PGF_2_α on LH-stimulated P4 secretion was significantly mitigated (Fig. 3). This observation has an important physiological corollary: both PGF_2_α-receptors and PKCε are expressed in the same luteal cell type. Therefore, the isozyme PKCε has an important compatible time (mid-luteal phase) and place (small and large luteal steroidogenic cells) of expression, for it to have a role in the luteal transition from resistance to sensitivity to luteolytic actions of PGF_2_α. Furthermore, if PKCε expression is down -regulated (this study) or if its activation is inhibited [27], the anti-steroidogenic effect of PGF_2_α on LH-stimulated P4 secretion is impaired.

Experiment 2 also tested the hypothesis that down-regulating PKCε could influence the expression of key PG metabolizing enzymes that, in turn, could influence the balance of PG production from luteo-protective or luteotrophic to luteolytic. The mechanism for luteal resistance is not exactly known. However there is now evidence that regulation of key PG metabolizing enzymes observed during physiological states in which the life span of the CL is modified is likely to play an important role in this complex process [41-49]. The selection of the examined genes was based on the available evidence that, because of their key positions in the PG biosynthetic pathway, these genes have been shown to determine the accumulation of luteolytic or luteotrophic classes of PG [40-45]. For example, we examined the effects of down-regulating PKCε on the expression of PGE_2 _and F synthases because of their more direct effect on determining whether PGH2 is metabolized to PGE_2 _or PGF_2_α. The results obtained were unexpected; the prediction was that because of low expression of PKCε, exogenous PGF_2_α would not be able to induce high increases in the cytosolic concentration of calcium, and consequently, the expression of PGE_2 _synthase/PGF_2_α synthase ratio would favor PGE_2 _synthesis. The above conditions would favor luteal function. However, it is worth pointing out the importance of looking beyond steady states of mRNA encoding these enzymes; sometimes regulation may be at the level of protein or even enzyme activity and additional work is necessary before rejecting the tested hypothesis.

The developmental significance of a regulatory role played by cytosolic calcium concentrations in mediating the inhibitory actions of PGF_2_α is documented by results obtained in experiment 3. As reported in previous studies [17], PGF_2_α reduced LH-stimulated P4 secretion in Day-10 cells only. Basal P4 secretion was not affected by the PGF_2_α-treatment at any of the two developmental stages tested. As the working hypothesis predicted, the pharmacological increase in [Ca^2+^]i induced by A23187 effectively mimicked the inhibitory effect of PGF_2_α in Day -10 steroidogenic cells. Furthermore, as predicted by the working hypothesis, the A23187 treatment also inhibited LH-stimulated P4 secretion in Day -4 steroidogenic cells. This inhibitory effect of A23187 is most likely due to its demonstrated effect in increasing the intracellular concentration of calcium ions [27] in these cells and not due to other non-specific effects. This interpretation is also supported by the observation that treatment with A23187 had no negative effect on basal P4 secretion at any of the two developmental stages tested.

Further support for the significance of a regulatory role played by the increase in [Ca^2+^]i in mediating the inhibitory actions of PGF_2_α is documented by results obtained in experiment 3 where the cytoplasmic calcium buffering capacity of the cells was increased by Bapta-AM. At lower concentrations (0.1 and 1.0 μmol), the calcium buffering capacity of Bapta-AM was, most likely, at values that still allowed a PGF_2_α-stimulated increase in [Ca^2+^]i; which in turn, preserved the ability of PGF_2_α to inhibit LH-stimulated P4 secretion (Fig. 6). However, as the calcium buffering capacity in the cytoplasm of the steroidogenic cells was increased by increasing the concentration of Bapta-AM (10 and 100 μmol), the calcium signaling feature of activating the PGF_2_α receptors was most likely eliminated or at least reduced, and consequently, the ability of PGF_2_α to inhibit LH-stimulated P_4 _secretion was also significantly reduced (Fig. 6). Similar effects of Bapta-AM on basal and hormonal-stimulated steroidogenesis have been reported in MA-10 Leydig cells (34). Therefore, the results of experiment 3 stress the calcium requirement for PGF_2_α to inhibit LH-stimulated P_4 _secretion in the mid-phase CL and support the reported observation that the lower efficacy of PGF_2_α to inhibit P_4 _secretion in the early CL is related to the reduced ability of PGF_2_α to increase the cytoplasmic concentration of calcium at this developmental stage [17]. Taken together, the results obtained in the A23187 and Bapta-AM experiments, strongly support the proposed hypothesis that an attenuation of the luteolytic actions of PGF_2_α is associated with a compromise in the ability of PGF_2_α to induce a rise in [Ca^2+^]i [27]. Therefore these studies provide a strong linkage between the signal transduction utilized by the PGF_2_α receptor at different developmental stages and quantitative aspects of the known intracellular mediator of PGF_2_α actions in the CL, [Ca^2+^]i. In this regard, species differences do exist, as in rat luteal cells the antigonadotropic action of PGF_2_α is not mediated by elevated cytosolic calcium levels [50]. It appears that the bovine CL therefore, has the following commonalities with human CL: 1) in both species, PGF_2_α is luteolysin, 2) the luteolytic effect of PGF_2_α appears only during mid- and late-luteal phase, and 3) in both, the humans and cows, changes in intracellular calcium appear to regulate luteal function ([51] and this study).

In summary, the evidence presented here strongly supports the idea that PKCε, an isozyme highly expressed in steroidogenic luteal cells with acquired luteolytic response to PGF_2_α, has an important regulatory role in the ability of PGF_2_α to inhibit LH-stimulated P4 secretion in vitro at this developmental stage. The data presented strongly support the hypothesis that luteal resistance to the luteolytic actions of PGF_2_α is associated with a compromised ability of PGF_2_α to induce a rise in [Ca^2+^]i. If the PGF_2_α receptor and its associated signal transduction is bypassed with a pharmacological agent to increase the [Ca^2+^]i, the LH-stimulated P4 secretion in Day -4 steroidogenic cells is eliminated, an action that cannot be induced by PGF_2_α at this developmental stage. Conversely, if the increase in [Ca^2+^]i typically induced by PGF_2_α on Day-10 steroidogenic luteal cells is buffered by a pharmacological agent, then the ability of PGF_2_α to inhibit the LH-stimulated P4 secretion is abrogated.

## Authors' contributions

MPG and AS made equal contributions to this study. MPG and AS were responsible for surgical procedures, all aspects of laboratory procedures, participated in the discussion, interpretation of results and in drafting the manuscript. EKI participated in the surgeries, design of the study, data analysis and drafting of the manuscript. JAF directed and participated in all aspects of the studies. All authors read and approved the final manuscript.
